# Disturbance in Maternal Environment Leads to Abnormal Synaptic Instability during Neuronal Circuitry Development

**DOI:** 10.3389/fnins.2017.00035

**Published:** 2017-02-06

**Authors:** Yusuke Hatanaka, Tomohiro Kabuta, Keiji Wada

**Affiliations:** ^1^Department of Degenerative Neurological Diseases, National Institute of Neuroscience, National Center of Neurology and PsychiatryTokyo, Japan; ^2^Department of Neurology, Graduate School of Medicine, Kyoto UniversityKyoto, Japan

**Keywords:** dendritic spines, synaptic development, maternal obesity, maternal hyperandrogenism, neuropsychiatric disorders

## Abstract

Adverse maternal environment during gestation and lactation can have negative effects on the developing brain that persist into adulthood and result in behavioral impairment. Recent studies of human and animal models suggest epidemiological and experimental association between disturbances in maternal environments during brain development and the occurrence of neuropsychiatric disorders, including autism spectrum disorder, attention deficit hyperactivity disorder, schizophrenia, anxiety, depression, and neurodegenerative diseases. In this review, we summarize recent advances in understanding the effects of maternal metabolic and hormonal abnormalities on the developing brain by focusing on the dynamics of dendritic spine, an excitatory postsynaptic structure. We discuss the abnormal instability of dendritic spines that is common to developmental disorders and neurological diseases. We also introduce our recent studies that demonstrate how maternal obesity and hyperandrogenism leads to abnormal development of neuronal circuitry and persistent synaptic instability, which results in the loss of synapses. The aim of this review is to highlight the links between abnormal maternal environment, behavioral impairment in offspring, and the dendiric spine pathology of neuropsychiatric disorders.

## Introduction

The maternal environment is inevitably an environmental factor that impacts brain development in most animals are born to a mother. Recent studies of human and animal models provide evidence that disturbances in the maternal environment during development are associated with many neuropsychiatric disorders, such as autism spectrum disorder (ASD) (Baron-Cohen et al., 2005; Patterson, 2011; Xu et al., 2013), attention deficit hyperactivity disorder (ADHD) (Ray et al., 2009), anxiety (Sullivan et al., 2010), depression (Rice et al., 2007), schizophrenia (Kawai et al., 2004), Alzheimer's disease (Lahiri et al., 2009), and Parkinson's disease (Barlow et al., 2007). In this mini-review, we focus on abnormal metabolic and hormonal conditions that affect mothers, and review the association between these maternal environments and offspring behavior. Because several neuropsychiatric disorders show large deficits in synaptic connectivity (Penzes et al., 2011), we later summarize the synaptic pathologies of developmental disorders and neurological diseases. We also introduce our recent studies that demonstrate how maternal high-fat diet (HFD) and hyperandrogenism duce abnormal development of neuronal circuitry in offspring, which results in the loss or excess of synapses in later life. Our studies link abnormal maternal environment-induced behavioral impairment in offspring with the dendritic spine pathology of neuropsychiatric disorders. Based on their shared synaptic pathology during the development of neuronal circuitry, we propose that many neuropsychiatric disorders have a common underlying deficit in synaptic development. Not only is this synaptic instability found in developmental disorders of the nervous system, it is also found in late-onset neurodegenerative diseases.

## Maternal environment is associated with neuropsychiatric disorders

### Maternal obesity

Obesity is a worldwide health problem and a major contributor to the increased incidence of coronary artery disease, hypertension, and Type-II diabetes (Kopelman, 2000). Due to factors listed, many women are obese or overweight by the time they reach childbearing age (Kanagalingam et al., 2005; Huda et al., 2010). Thus, maternal obesity can be a major contributor to the disturbance in brain development of the children during pre- and postnatal period. Epidemiological studies have shown that maternal obesity has adverse effects on brain development in children, which can result in ADHD (Rodriguez et al., 2008; Rodriguez, 2010), schizophrenia (Kawai et al., 2004), cognitive impairments (Van Lieshout et al., 2011), and eating disorders (Favaro et al., 2006; Allen et al., 2009). Maternal obesity also predisposes children to metabolic disorders (Deierlein et al., 2011), and is associated with depression (Rofey et al., 2009), anxiety disorder (Rofey et al., 2009), learning disability (Cserjési et al., 2007), ADHD (Waring and Lapane, 2008), and Alzheimer's disease (Luchsinger et al., 2002). Additionally, animal models have demonstrated the negative effects of maternal obesity on the brain and peripheral organs of offspring (Williams et al., 2014). We previously reported that mouse pups from obese dams fed with a HFD show peroxidized lipid accumulation in many brain regions, impaired adult neurogenesis in the hippocampus, and deficits in spatial learning performance (Tozuka et al., 2009, 2010). Other animal-model studies have also demonstrated the impairments in conditioned and reversal learning in offspring from HFD-fed dams (Rodriguez et al., 2012; Wu et al., 2013). These lines of evidence suggest that maternal HFD consumption and obesity impact brain development of the offspring, resulting in behavioral impairments in adulthood. Accumulating evidence suggest that maternal HFD consumption leads to the perturbations in serotonergic and dopaminergic systems that regulate brain function and development (Sullivan et al., 2010; Vucetic et al., 2010). Increased levels of inflammatory cytokines (Das, 2001), insulin (Leung and Lao, 2000), and leptin (Lepercq et al., 2002) in obese mothers are considered to influence the development of these neurotransmitter signaling pathways. However, neuronal circuitry and molecular mechanisms underlying the association between maternal HFD consumption and abnormal behavior of the children remain to be elucidated.

### Maternal hormonal abnormality

Human and animal-model studies have demonstrated an association between prenatal *in utero* exposure to testosterone and the deficits in social interaction that was diagnosed as ASD (Baron-Cohen et al., 2005; Hines, 2011; Xu et al., 2013, 2015). Sex steroids such as androgens and estrogens shape the structure of sexual dimorphisms during fetal brain development by their organizing effects (Phoenix et al., 1959; Keefe, 2002; Bonthuis et al., 2010). Intrauterine levels of steroid hormones, including stress and sex steroids, are directly affected by the physical condition of the mother. For example, psychological stress in women correlates with the concentration of testosterone in the blood (Chichinadze and Chichinadze, 2008; Lennartsson et al., 2012). Furthermore, maternal stress during pregnancy is associated with increased risk of ASD in children (Keefe, 2002). Polycystic ovary syndrome (PCOS), characterized by ovarian dysfunction, and polycystic ovarian morphology in women of childbearing age, leads to excessive levels of male hormones, a condition called hyperandrogenism (Franks, 1995; Abbott et al., 2005; Azziz et al., 2005; Goodarzi et al., 2011), and children from PCOS mothers are thought to express more autistic traits (Palomba et al., 2012). Indeed, an animal-model study of maternal hyperandrogenism during pregnancy demonstrated that the offspring exhibit autistic-like behavior from adolescence to adulthood (Xu et al., 2015). These lines of evidence suggest that prenatal exposure to testosterone leads to developmental deficits of the brain, resulting in abnormal behavior; however, the underlying mechanism of development of autistic-like behavior remains unclear.

## Neuropsychiatric disorders and their dendritic spine pathologies

The synapse plays an essential role in brain function. Dendritic spines are postsynaptic structures that receive glutamatergic excitatory input from presynaptic terminals. Dynamic changes in the density and morphology of dendritic spines are associated with the rewiring of neuronal circuits during circuit development (Grutzendler et al., 2002), experience-dependent neuronal plasticity (Trachtenberg et al., 2002), aging (Mostany et al., 2013), and the progression of many neuropsychiatric diseases (Penzes et al., 2011). Human postmortem studies have revealed higher than normal cortical spine density in ASD (Hutsler and Zhang, 2010) and fragile X syndrome (Irwin et al., 2001), which is thought to the result from deficiencies in synaptic pruning during the development of neuronal circuitry. In contrast, spine density was shown to be markedly lower in the postmortem brains of people who had Alzheimer's disease (Tackenberg et al., 2009) or schizophrenia (Selemon and Goldman-Rakic, 1999). Numerous proteins associated with these disorders are involved in the regulation of dendritic spine density and morphology (Penzes et al., 2011).

Recent animal studies using *in vivo* two-photon imaging have suggested that dendritic spine instability results in the aberrant spine density and morphology observed in the brains of mouse models for fragile X syndrome (Cruz-Martín et al., 2010), ASD (Isshiki et al., 2014), and schizophrenia (Hayashi-Takagi et al., 2014). Dendritic spine instability is thought to be associated with abnormal rewiring of neuronal circuits, a phenomenon also present in neurodegenerative diseases such as Alzheimer's disease (Tsai et al., 2004) and Huntington's disease (Murmu et al., 2013). The dendritic spine instability is assessed by the elevation of formation and elimination rate of spines, and the imbalance between formation and elimination rate leads to subsequent loss or excess of spines. These lines of evidence suggest that dendritic spine instability might be a common pathology to both neurodevelopmental disorders and neurodegenerative diseases. Using a knock-in model mouse that faithfully replicates human symptomatic and pathological features of spinocerebellar ataxia type 1 (SCA1), a late-onset polyglutamine neurodegenerative disease characterized by ataxia, cognitive impairment, and neuronal death, we previously demonstrated that dendritic spine instability becomes evident before pronounced motor incoodination (Hatanaka et al., 2015b). Interestingly, this abnormal synaptic instability is present even during synaptic development. In normal development, dendritic spines are initially unstable, and they become stable in the adult (Grutzendler et al., 2002). Thus, immature dendritic spines during development may more vulnerable to internal and external factors than mature spines. These lines of evidence suggest that a latent developmental abnormality in neuronal circuitry might subsequently lead to neuronal dysfunction and loss in late-onset neurodegenerative diseases, which have otherwise been believed to begin during middle age.

## Disturbance in maternal environment impacts dendritic spine dynamics during synaptic development

Abnormal maternal environments are associated with several neurodevelopmental disorders and neurodegenerative diseases that are characterized by very early synaptic impairment. However, the synaptic mechanism that translates certain maternal environments into future behavioral impairments in offspring is not fully understood. Recently, we reported that maternal HFD consumption and excess testosterone exposure during brain development leads to persistent synaptic instability in mouse offspring (Hatanaka et al., 2015a,b). In this section, we review these two studies and discuss the synaptic mechanism.

### Maternal HFD consumption and synaptic impairment in offspring

Studies of human and animal models have demonstrated that maternal obesity negatively impacts the neurodevelopment of children (Williams et al., 2014). Mouse pups from obese dams fed with an HFD show peroxidized lipid accumulation in many brain regions, impaired adult neurogenesis in the hippocampus, and deficits in spatial learning, conditioning, and adaptation (Tozuka et al., 2009, 2010; Rodriguez et al., 2012; Wu et al., 2013). However, the synaptic basis for maternal HFD-induced brain dysfunction has remained unclear. Peroxidized lipid accumulation in the brains of the offspring from HFD-fed dams disappears when the offspring are raised on a normal diet after weaning, whereas abnormal progenitor-cell proliferation in the hippocampus and ADHD-like hyperactivity persist into adulthood (Tozuka et al., 2009, 2010). Thus, there may be reversible and irreversible components of the maternal HFD-induced brain impairment found in offspring. To determine the critical period that is most susceptible to maternal HFD consumption for the synaptic impairment, and to establish the association between the oxidative stress in this period (which results from peroxidized lipid accumulation) and impairment in neuronal circuitry development, we analyzed the dynamics and morphology of dendritic protrusions using *in vivo* two-photon laser-scanning microscopy. This analysis must be conducted *in vivo* because peripheral organs and non-neuronal cells of the offspring exhibit abnormal metabolic homeostasis that contributes a great deal to synaptic function (Bilbo and Tsang, 2010; Williams et al., 2014).

By analyzing the formation and elimination rate of dendritic spines, we have found that maternal HFD leads to instability of spines in the cerebral cortex of juvenile offspring, even when they are fed with a normal diet after weaning (Hatanaka et al., 2016). This effect persists into adulthood and manifests as a decline in dendritic spine number. When offspring are exposed to maternal HFD exclusively during lactation, their synaptic instability and loss of spines is greater than or equal to that in offspring who are exposed to pre- and postnatal maternal HFD. Antioxidant-treatment during lactation ameliorates the synaptic impairment in the offspring born to HFD-fed dams. These results suggest that maternal obesity causes sustained synaptic impairments in offspring, which may be associated with brain dysfunction in adulthood, and that these impairments may result from oxidative stress caused by peroxidized lipid accumulation during lactation. The maternal metabolic milieu is one of the most important factors that impacts the development of neuronal circuitry and brain function in adulthood. Using *in vivo* imaging while maintaining the intravital environment, this study provided the first evidence for the synaptic basis of the brain dysfunction in offspring of obese dams.

### Prenatal testosterone exposure and abnormal synaptic development of offspring

ASD is a neurobehavioral syndrome with a heterogeneous phenotype, and its overall prevalence is about 1/100 (Fernell and Gillberg, 2010). Men are more likely to be affected than women, with a 4:1 ratio (Freitag, 2007; Abrahams and Geschwind, 2008). Many lines of evidence from human and animal studies suggest that disturbances in synaptic homeostasis may be a key factor in the development of ASD (Toro et al., 2010; Delorme et al., 2013). Although the pathogenesis of ASD is most likely polygenic and potentially epistatic, maternal environmental factors might also interact with genetic factors to increase risk (Gardener et al., 2011; Hallmayer et al., 2011). Epidemiological and animal-model studies suggest that abnormal prenatal exposure to testosterone results in autistic-like behavior in the children (Baron-Cohen et al., 2005; Hines, 2011; Xu et al., 2013). However, the synaptic pathogenesis of abnormal behaviors exhibited by children who are prenatally exposed to excess testosterone remains unexplained. Because neuronal circuitry maturation is accomplished by dendritic spine stabilization and pruning (Grutzendler et al., 2002), we analyzed dendritic spine stabilization during synaptic development in the mouse offspring from PCOS model dams, as a means of estimating deficiency in neuronal circuitry development. Rodent models of PCOS are administered testosterone prenatally and exhibit autistic-like behavior, infertility, obesity, hyperinsulinism, an increased risk of type-II diabetes, cardiovascular disease, and other abnormal reproductive and metabolic functions (Walters et al., 2012). Thus, analyzing dendritic spines *in vivo* is important because it preserves the contributions that peripheral tissues provide to spine dynamics.

By using *in vivo* two-photon imaging, we have found that mice exposed prenatally to testosterone show increased rates of spine formation and elimination in the frontal cortex at the developmental stage of synapse formation (4-week-old), and that this synaptic instability persists into adulthood (8-week-old) (Hatanaka et al., 2015a). Density of dendritic spines is excessively high, and their morphology is abnormal even when they are in adulthood. This is consistent with post-mortem studies of ASD and fragile X syndrome (Irwin et al., 2001; Hutsler and Zhang, 2010) and *in vivo* studies of animal models for these conditions (Cruz-Martín et al., 2010; Pan et al., 2010; Jiang et al., 2013; Isshiki et al., 2014). These results suggest that synaptic instability, excess density, and abnormal morphology of dendritic spines are the synaptic basis of subsequent neurodevelopmental deficits in prenatally testosterone-exposed mice that exhibit autistic-like behavior. This study was the first to examine the synaptic basis of neurodevelopmental impairments in offspring who were prenatally exposed to a hyperandrogenic environment.

## Conclusion

**Figure 1:**
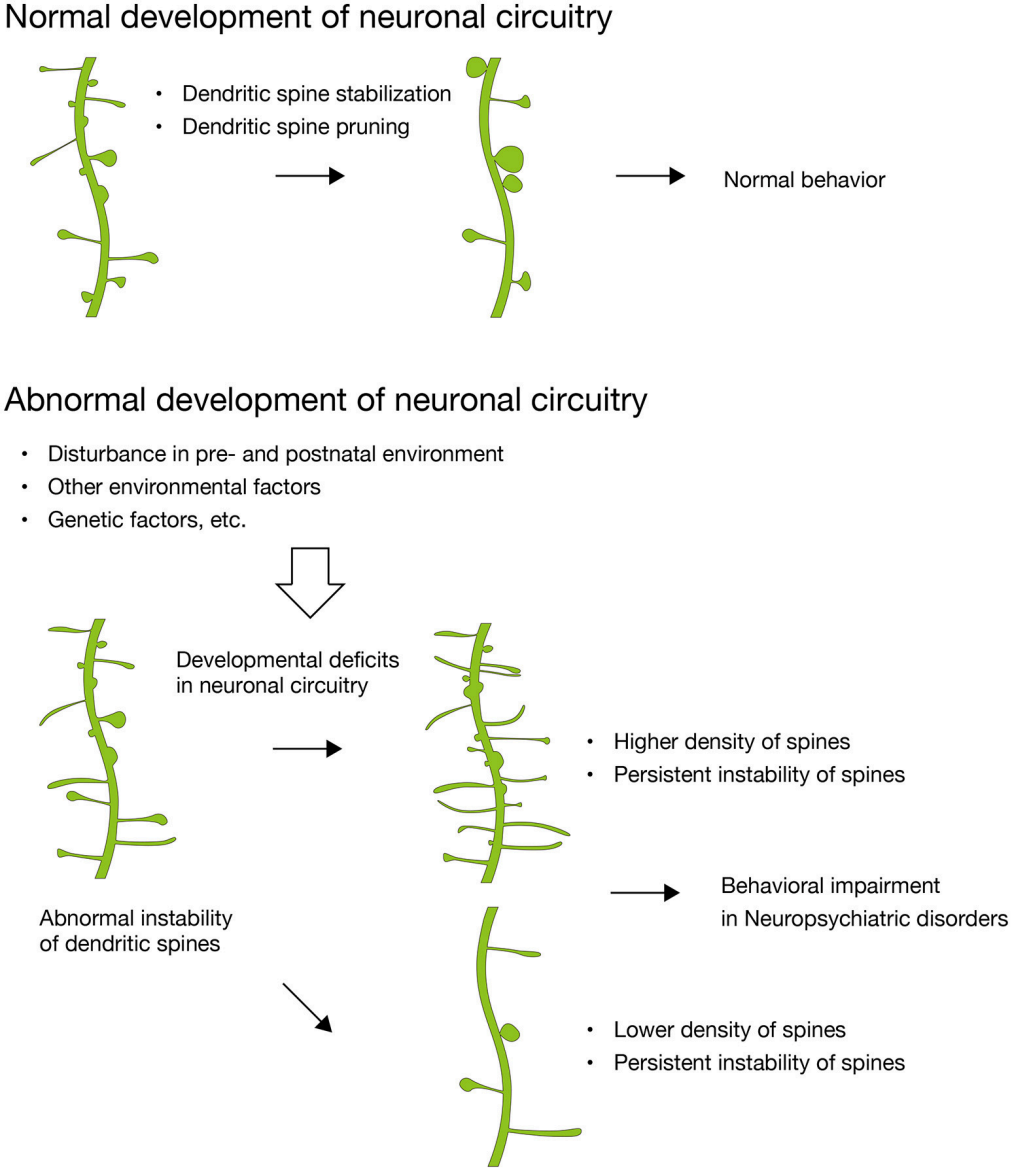
**Abnormal synaptic instability during the development of neuronal circuitry**. In normal development, dendritic spines are stabilized and pruned with maturation. In contrast, dendritic spines in many neuropsychiatric disorders show abnormal instability, which results in higher or lower density of dendritic spines and divergent behavioral impairments subsequently. Disturbance in maternal environment critically impact on dendritic spines of the fetal brain and leads to developmental deficits in neuronal circuitry (Hatanaka et al., 2015a,b, 2016).

Recent studies have shown that disturbances in maternal environment are related to neuropsychiatric disorders. However, details regarding the synaptic basis for this phenomenon remain an open question. In this review, we focused on abnormal synaptic instability during synaptic development, which results in excess or loss of synapses in adulthood and in behavioral impairments (Figure 1). We summarized recent discoveries of dendritic spine pathologies in many neurodevelopmental disorders and neurodegenerative diseases that are associated with disturbances in maternal environment, and propose that the abnormal synaptic instability observed during development of neuronal circuitry is a shared pathology of these neuropsychiatric disorders. The evidence suggests that deficits in synaptic development might be a pathology shared among many neuropsychiatric disorders, including both developmental disorders and late-onset neurodegenerative diseases. Although synaptic instability is a common deficit among these neuropsychiatric disorders, the symptoms and their onset of these different disorders are highly diverse. This can be explained by the neuronal circuitry specificity impaired in each disorder, and the specificity is due to the expression profiles of disease-associated genes. Further studies that combine genetic and environmental risk factors are needed in order to explain the diversity of the neuropsychiatric disorders. Furthermore, additional comprehensive studies demonstrating the relationship between disturbances in maternal environmental, abnormal synaptic instability during neuronal circuitry development, and behavioral impairments in offspring are necessary for further understanding the synaptic mechanisms underlying developmental disorders in the brain.

## Author contributions

YH, KW, and TK wrote the manuscript.

## Funding

This work was supported by Intramural Research Grants for Neurological and Psychiatric Disorders, NCNP [to TK], the Japan Society for the Promotion of Science, Grants-in-Aid for Young Scientists (B) [25871174 and 16K19508 to YH], and a grant from Core Research for Evolutional Science and Technology (CREST), Japan Science and Technology Agency (JST) [to KW].

### Conflict of interest statement

The authors declare that the research was conducted in the absence of any commercial or financial relationships that could be construed as a potential conflict of interest.
